# Supplementary material for: The Cytogenetic Map of the Nile Crocodile (Crocodylus niloticus, Crocodylidae, Reptilia) with Fluorescence In Situ Localization of Major Repetitive DNAs
Source: Int J Mol Sci. 2022 Oct 27;23(21):13063. doi: 10.3390/ijms232113063 (PMC9656864; doi:10.3390/ijms232113063)
Supplement: Supplementary file 1 [file ijms-23-13063-s001.zip › ijms-1918366-supplementary.pdf]

Table S1. Primers designed with Gene Runner software (version 6.5.52) produced by Dr. Spruyt Michael (Mahwah, NJ, USA) and Dr. Buquicchio Frank (Old Tappan, NJ, USA) for PCR amplification and labeling of repetitive probes. For short sequences, primers were matched to several copies of the monomer.

**Repetitive sequence name**	**F-primer**	**R-primer**	**Product length**	**Product GC-content**	**Primer melting temperature**
CNI-Sat-4	CCCATTCGCTTGCCTG	AGCCACCAGGTCAACCC	73	64.38	58.97
CNI-Sat-19	GTTTAAAGTGGTTTGCAACTGC	ACTGGTTGAAACAGCTTCTGG	75	42.67	53.29
CNI-Sat-36	GAGGCCGGAAATCCCTTC	GAGCCGAGTCCTCCGAAAC	48	54.17	53.71
CNI-Sat-58	TCCAGTTCTGCATATCTCCACC	AGAGCTGGAGATATTCAGAGCG	73	43.84	53.80
CNI-Sat-67	TTGCAAGCAGTTTAGACCAGTC	GCGTTAGCAAAGCACTTTAAAC	51	43.14	50.45
CNI-Sat-93	GCGCGGTCTGGTTGTG	CGCACACACACACGCAC	92	67.39	61.18
CNI-Sat-96	CAGTTAGAACTGGTTTGGACTG	CAGTATAAACCAGCTTAAACCG	132	37.88	53.85
CNI-LTR-48	GCACAAAGGCGGTTCGTTTC	GTTCCTGTTTCTGCACACGTGC	186	47.85	60.30
CNI-LTR-68	CTAACCCTGCAACAGCGAGATC	ATGGAAGACCACGAGTGTCGAG	275	54.91	62.71
